# Supplementary material for: Immune Response and Serum Biomarker Screening in Pregnant Women with Influenza A Virus Infection: Insight into Susceptibility and Disease Severity
Source: Diseases. 2025 Jun 10;13(6):182. doi: 10.3390/diseases13060182 (PMC12192047; doi:10.3390/diseases13060182)
Supplement: Supplementary file 1 [file diseases-13-00182-s001.zip › diseases-3619615-supplementary.pdf]

Supplemetary Table S1. Reagents used in this study.

Reagents name	Brand	Cat no.
Ficoll-Paque PREMIUM	GE Healthcare	17544203
DPBS	Gibco	C14190500BT
Red blood cell lysate	Beyotime	C3702
DAPI	Beyotime	C10072
FITC Anti human CD3 antibody	Biolegend	317306
APC Anti human CD4 antibody	Biolegend	300514
PerCP Cy5.5 Anti human CD8 antibody	Biolegend	344710
APC Cy7 Anti human CD16 antibody	Biolegend	302018
PE Anti human CD56 antibody	Biolegend	362508
APC anti human CD19 antibody	Biolegend	302212
FITC anti human CD27 antibody	Biolegend	356404
PE anti human CD38 antibody	Biolegend	303506
Th1/Th2/Th17 human CBA cytokine kit	BD Bioscience	560484
Influenza virus universal PCR kit	Zhijiang	RR-0097

Legends: Brand name and catalog number of the reagents are listed in this table. DPBS: Dulbecco's phosphate buffered saline; DAPI: 4',6-diamidino-2- phenylindole; CBA: Cytometric bead array. .

Supplementary Table S2. The demographic and clinical data of the participants.

Groups	CN n=37	PN n=133	PH3 n=62	PSI n=20
**Age (Year)**	28.7±3.54	29.4±4.86	28.6±4.58	29.9±4.15
**Gestation**				
No-pregnancy	37 (100%)	0 (0.0)	0 (0.0)	0 (0.0)
Trimester 1st	0 (0.0)	45 (33.83%)	12 (19.4%)	0 (0.0)
Trimester 2nd	0 (0.0)	49 (36.84%)	11 (17.7%)	0 (0.0)
Trimester 3rd	0 (0.0)	39 (29.32%)	39 (62.9%)	20 (100%)
**Sympotome**				
Fever (>37.3℃)	0 (0.0)	0 (0.0)	57 (91.9)	19 (95.0)
pharyngalgia	0 (0.0)	0 (0.0)	36（58.1）	14(70.0)
cough	0 (0.0)	0 (0.0)	48（77.4）	15（75.0）
Expectoration	0 (0.0)	0 (0.0)	29（46.8）	9（45.0）
Dyspnea	0 (0.0)	0 (0.0)	6（9.7）	2（10.0）
**Vaccination in the past year**				
Sensonal influenza vaccine	0 (0.0)	0 (0.0)	0 (0.0)	0 (0.0)
Haemophilus influenza type B vaccine	0 (0.0)	0 (0.0)	0 (0.0)	0 (0.0)
Exposure history to influenza	0 (0.0)	0 (0.0)	25（40.3）	8（40.0）
**Influenza related serious outcomes**				
Oxygen inhalation	0 (0.0)	0 (0.0)	0 (0.0)	20（100.0）
mechanical ventilation	0 (0.0)	0 (0.0)	0 (0.0)	0 (0.0)
ICU	0 (0.0)	0 (0.0)	0 (0.0)	0 (0.0)
**Comorbidities**				
Autoimmune disease	0 (0.0)	0 (0.0)	0 (0.0)	0 (0.0)
Pneumonia	0 (0.0)	0 (0.0)	0 (0.0)	2 (10%)
Acute respiratory distress syndrome (ARDS)	0 (0.0)	0 (0.0)	0 (0.0)	1(5%)
Respiratory failure	0 (0.0)	0 (0.0)	0 (0.0)	0 (0.0)
Septic shock	0 (0.0)	0 (0.0)	0 (0.0)	0 (0.0)
Disseminated intravascular coagulation (DIC)	0 (0.0)	0 (0.0)	0 (0.0)	0 (0.0)

**Hospital level for consultation**				
Level 1	0 (0.0)	0 (0.0)	0 (0.0)	0 (0.0)
Level 2	0 (0.0)	0 (0.0)	7（11.3）	1（5.0）
Level 3	37 (100%)	133 (100%)	55（88.7）	19（95.0）

Legends: This table shows the demographic and clinical data of the participants in the four groups. Data are shown as mean±SD in age, and participant numbers (percentage of this group) in other indexes.

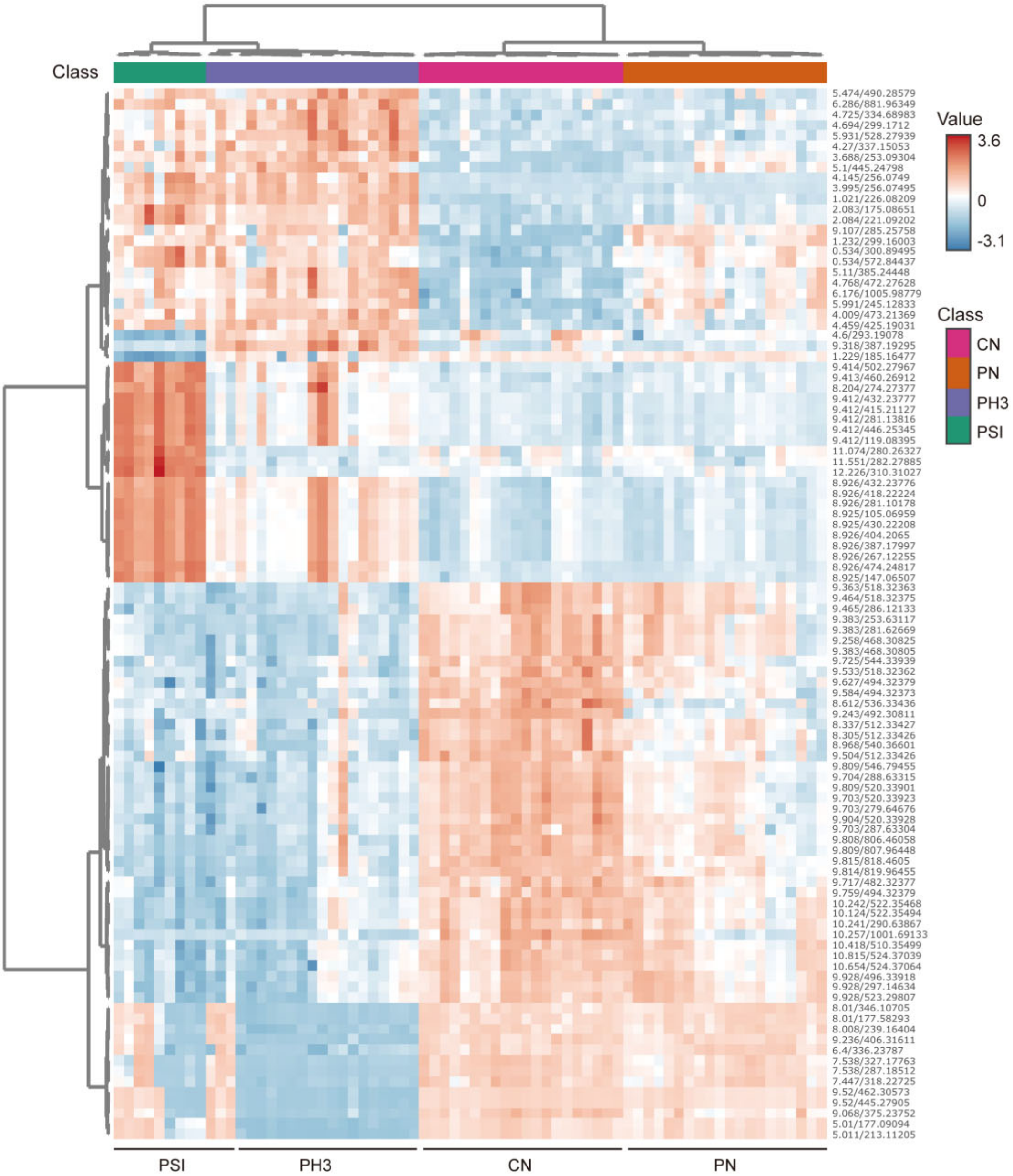

Supplementary Figure S1. Heatmap of the differential metabolites. The top 100 metabolites among the four groups were enriched. The metabolite abundance was normalized by z score across samples. The red dots represent high expression as to 3.6, and the blue dots represent low expression as to -3.1. m/z of each metabolite was shown in the right.
